# Perovskite in Triboelectric Nanogenerator and the Hybrid Energy Collection System

**DOI:** 10.3390/ma17236019

**Published:** 2024-12-09

**Authors:** Tong Wu, Zequan Zhao, Yin Lu, Hanzhang Yang, Xiaoning Liu, Xia Cao, Ning Wang

**Affiliations:** 1 Center for Green Innovation, School of Mathematics and Physics, University of Science and Technology Beijing, Beijing 100083, Chinam202410799@xs.ustb.edu.cn (H.Y.);; 2 National Institute of Metrology, Beijing 100029, China; 3 Beijing Institute of Nanoenergy and Nanosystems, Chinese Academy of Sciences, Beijing 100083, China; 4 School of Chemistry and Biological Engineering, University of Science and Technology Beijing, Beijing 100083, China

**Keywords:** triboelectric nanogenerators, perovskite materials, optoelectronic properties, energy conversion

## Abstract

In the context of escalating energy demands and environmental sustainability, the paradigm of global energy systems is undergoing a transformative shift to innovative and reliable energy-harvesting techniques ranging from solar cells to triboelectric nanogenerators (TENGs) to hybrid energy systems, where a fever in the study of perovskite materials has been set off due to the excellent optoelectronic properties and defect tolerance features. This review begins with the basic properties of perovskite materials and the fundamentals of TENGs, including their working principles and general developing strategy, then delves into the key role of perovskite materials in promoting TENG-based hybrid technologies in terms of energy conversion. While spotlighting the coupling of triboelectric–optoelectronic effects in harnessing energy from a variety of sources, thereby transcending the limitations inherent to single-source energy systems, this review pays special attention to the strategic incorporation of perovskite materials into TENGs and TENG-based energy converting systems, which heralds a new frontier in enhancing efficiency, stability, and adaptability. At the end, this review highlights the remaining challenges such as stability, efficiency, and functionality for applications in TENG-based energy-harvesting systems, aiming to provide a comprehensive overview of the current landscape and the prospective trajectory of the role of perovskite materials in TENG-based energy-harvesting technologies within the renewable energy sector.

## 1. Introduction

The current global energy landscape is marked by a pressing and complex challenge: meeting the escalating demand for power while mitigating the environmental impact of energy generation [1]. This situation has emerged as a critical concern, primarily driven by the rapidly growing global population and the relentless pace of industrialization. Traditional energy sources, mainly fossil fuels, while being the mainstay of the global energy supply, are increasingly unsustainable due to their finite nature and significant contribution to environmental degradation, notably climate change. This reality has necessitated a shift toward more sustainable, reliable, and eco-friendly energy-harvesting methods [2,3,4]. The urgent need for such transformative energy solutions is not only a response to the depletion of conventional energy reserves but also a reflection of the global commitment to reducing carbon footprints and combating the adverse effects of climate change [5,6]. The new types of green energy mainly include water energy, biomass energy, solar energy, wind energy, and ocean energy [7,8,9,10,11].

Solar energy, harnessed from the sun’s abundant and inexhaustible supply, has emerged as a leading solution in this quest for sustainable energy alternatives. Its potential to provide clean, renewable, and efficient energy makes it a cornerstone in the transition toward a greener energy future [12,13]. The advent of perovskite materials has further enhanced the prospects of solar energy harvesting [14,15]. Perovskites, with their exceptional light absorption properties, high charge carrier mobility, and versatile fabrication capabilities, have revolutionized solar cell technology [16]. They have enabled the creation of solar panels with higher efficiencies and lower production costs, making solar energy more accessible and economically viable [17]. However, the intermittent nature of solar energy, reliance on climatic conditions, and limitations in energy storage technologies pose obstacles to its consistent and reliable utilization [18,19].

For continuous energy harvesting and power supply, the scientific community has ventured into a myriad of approaches, yet the journey has been marked by modest advancements [20]. Traditional methods to augment solar energy efficiency and reliability often revolve around improving photovoltaic cell materials, enhancing energy storage solutions, and developing more effective solar tracking systems [21,22,23]. However, these approaches have been largely constrained by technological, economic, and environmental factors [24,25]. For instance, while advancements in photovoltaic materials have led to higher efficiencies, they often come at increased costs or involve materials that pose environmental risks [26]. Similarly, energy storage solutions, crucial for mitigating solar energy’s intermittency, remain hampered by cost, capacity, and longevity challenges [27]. The limitations of these methods illuminate the need for a groundbreaking solution—one that not only complements solar energy’s strengths but also compensates for its weaknesses [28,29].

It is in this context that innovative energy technologies such as TENGs offer a novel and promising complementary solution to solar cells and other renewable energy devices [30,31,32,33]. TENGs operate on the principles of triboelectric effect and electrostatic induction, enabling the conversion of mechanical energy from various mechanical energy sources into electrical energy. This unique capability positions TENGs as a versatile and efficient complement to solar energy systems. One of the most compelling attributes of TENG technology is its ability to harvest mechanical energy continuously, regardless of weather conditions or time of day, thus addressing one of the fundamental limitations of solar power [34,35,36,37]. Furthermore, TENGs can capture energy from a wide range of sources, including wind, water waves, and even human motion, making them adaptable to diverse environments and applications [38,39]. Their flexibility and durability add to their appeal, allowing for integration into various structures and surfaces.

Despite the promising capabilities of TENGs, like any emerging technology, they have their challenges and limitations. A primary concern with TENGs is their energy density. While it is efficient for small-scale applications, it may not meet the demands of larger, more energy-intensive applications [40,41]. Additionally, TENGs depend on mechanical motion for energy generation, which can be a limiting factor in environments with minimal kinetic activity [42,43,44,45].

When integrating TENGs with solar energy to create a hybrid energy-harvesting system, additional complexities arise. This innovative combination faces challenges in system integration, optimization, and energy management. The efficiency of the hybrid system hinges on effectively interfacing the solar and mechanical energy-harvesting mechanisms, a task that can be technically demanding. Moreover, the range of energy harvesting is potentially limited. Solar energy is primarily weather dependent, while the performance of TENGs is influenced mainly by the availability and intensity of mechanical movements [46,47]. These factors call for a meticulous consideration of the operational environment and the specific application of the hybrid system. The limitations and drawbacks of a TENG–solar hybrid system underscore the necessity for ongoing research and development. This effort is crucial to optimize performance and broaden the energy-harvesting capabilities of these systems [48,49,50].

The introduction of perovskite materials into TENG technology marks a significant stride forward in addressing the shortcomings, such as efficiency, of TENGs. Perovskites, known for their remarkable electronic and optical properties, have the potential to significantly enhance the performance of TENGs [13,18]. By integrating perovskite materials, scientists have successfully increased the charge density in TENGs, thereby improving their energy output. This enhancement is particularly beneficial in maximizing the efficiency of TENGs in low-mechanical-energy environments. Furthermore, the opto-coupling capabilities of perovskites have been leveraged to boost power generation in TENGs, effectively harnessing both mechanical and solar energies in hybrid systems [51]. This dual energy-harvesting approach results in a more consistent and reliable energy output, overcoming some of the intermittency issues associated with solar power. Another critical advantage of incorporating perovskites into TENGs is the improvement in their stability [34].

**Figure 1:**
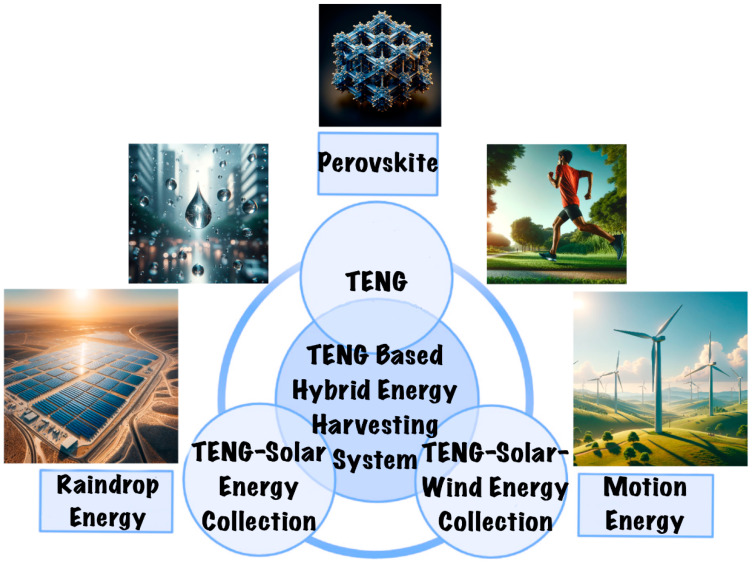
TENG-based hybrid energy-harvesting system.

Meanwhile, the concept of multihybrid energy-harvesting devices has emerged as a comprehensive solution to address the limitations of TENG–solar energy systems [52]. These advanced devices are designed to simultaneously collect energy from a diverse array of sources, including wind, bioenergy, solar energy, and even tidal movements (Figure 1) [53,54]. This multifaceted approach allows for the harnessing of energy in various environmental conditions, significantly expanding the scope of application [55,56]. For instance, a multihybrid device can capture solar energy during sunny days, wind energy in breezy conditions, and mechanical energy from human or vehicular movements, ensuring a constant and more reliable energy supply.

## 2. Photovoltaic and Triboelectric Effects

### 2.1. The Principle of the Photovoltaic Effect

**Figure 2:**
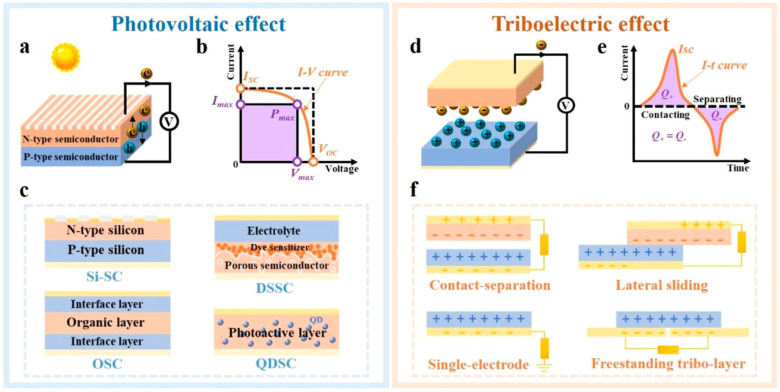
Photovoltaic and triboelectric effects: (**a**) Working principle. (**b**) I-V curve. (**c**) Different types of solar cells: (**d**) working principle, (**e**) I-t curve, and (**f**) working modes of TENGs [65]. Wu et al. (Elsevier, 2021).

Solar cells represent a cutting-edge technology in renewable energy, capable of transforming sunlight into electrical energy through the photovoltaic effect. This process occurs when solar radiation interacts with a semiconductor material within the solar cell, leading to the generation of electron–hole pairs and the consequent flow of electric current (Figure 2a). A critical measure of a solar cell’s performance is its power conversion efficiency (PCE), which essentially quantifies how effectively the cell converts incident solar energy into electrical energy. The PCE is calculated using the following formula:

(1) $$PCE\left(\%\right)=\frac{P_{max}}{P_{in}}=\frac{V_{OC}\times J_{SC}\times FF}{P_{in}\times 100}$$

In this equation, P_in_ represents the input solar energy, while V_OC_, J_SC_, and FF denote the open-circuit voltage, short-circuit current, and fill factor, respectively. The PCE is influenced by factors like the solar cell’s temperature and the spectrum and intensity of the incident sunlight. To ensure fair comparison among various solar cells, standardized testing conditions, typically under AM 1.5 at room temperature, are used.

The upper limits of voltage and current in solar cells are defined by V_OC_ and J_SC_. Interestingly, the power output is zero at these points because V_OC_ is only present without current flow, and J_SC_ occurs at zero voltage. The fill factor (FF) is crucial as it represents the ratio of the actual maximum obtainable power to the theoretical power defined by V_OC_ and J_SC_. Graphically, FF is depicted as the area of the largest rectangle that can fit within the I–V curve, indicative of the curve’s “squareness” (Figure 2b). The mathematical expression for FF is

(2) $$FF=\frac{P_{max}}{V_{OC}\times J_{SC}}=\frac{V_{max}\times J_{max}}{V_{OC}\times J_{SC}}$$

Solar cell technology has evolved through advancements in materials, device architecture, and fabrication techniques. As a result, various types of solar cells have been developed, including silicon-based cells (Si-SCs), dye-sensitized cells (DSSCs), organic solar cells (OSCs), quantum-dot solar cells (QDSCs), and perovskite solar cells (PSCs), each with distinct characteristics and applications (Figure 2c). Among these, all except PSCs have shown efficacy in harvesting light energy for high-performance triboelectric nanogenerators (HPTNGs).

### 2.2. The Principle of TENGs

TENGs are innovative devices that harness the triboelectric effect and electrostatic induction to transform biomechanical energy into electrical energy. The fundamental mechanism involves the transfer of electrons between materials of differing electronegativities during contact (Figure 2d). As these materials separate, an electrostatic induction mechanism triggers a flow of electrons toward an external circuit, resulting in the generation of alternating current through repetitive contact and separation actions (Figure 2e).

TENGs are generally divided into four main categories (Figure 2f): vertical contact-separation mode, lateral sliding mode, single-electrode mode, and free-standing triboelectric layer mode.

The vertical contact-separation mode [57,58] configuration involves two triboelectric substances with opposite electrical charges placed in close proximity. They periodically come into contact and move away from each other vertically. Contact leads to the generation of triboelectric charges at the interface, and their separation causes these charges to redistribute, creating an electrical potential that drives electrons across an external load, thus producing electricity.

In lateral sliding mode [59,60], here, two triboelectric substances slide horizontally over each other. This sliding motion generates triboelectric charges in a manner akin to the vertical mode. The relative parallel motion of these materials alters the area of overlap continuously, leading to a dynamic electric potential difference that propels electrons through an external circuit.

In single-electrode mode [61,62], only one of the triboelectric materials is connected to an electrode, while the other remains isolated. The isolated material is periodically brought into contact with and then separated from the electrode-bearing material. This action induces charges on the solitary electrode, and an electric potential difference between this electrode and a ground connection drives electrons through the external load, producing electrical energy.

In the freestanding triboelectric layer mode [63,64] setup, a triboelectric layer that stands freely is placed between two electrodes. This layer exhibits different triboelectric charges on each side. The electrodes periodically make and break contact with this layer. The resulted deformation generates triboelectric charges at the interfaces between the layer and the electrodes, and the resultant electric potential difference encourages electron flow through the external load.

## 3. Perovskite-Based TENGs

TENGs have emerged as a revolutionary technology for harvesting mechanical energy and converting it into electrical energy. The integration of perovskite materials into TENGs represents a significant leap forward in this domain, offering enhanced electrical output, stability, and efficiency. Perovskites, known for their remarkable electrical, optical, and physical properties, have opened new avenues for optimizing TENG performances in various applications, from self-powered devices to sustainable energy solutions.

### 3.1. High Charge Density of Perovskite-Based TENGs

The quest to improve the charge density of TENGs has led to the exploration of perovskite materials. Perovskites’ unique structural and electronic properties make them ideal candidates for enhancing the charge density in TENGs. Their ability to alter electronic and ionic conductivities, along with tunable surface properties, provides a pathway to significantly boost the efficiency and output of TENGs.

#### 3.1.1. Fully Inorganic Perovskite to Improve the Output of TENGs

The introduction of fully inorganic perovskites in TENGs marks a critical step in enhancing their electrical output. These perovskites, characterized by their stability and high charge-carrier mobility, contribute to an increased surface charge density and improved energy conversion efficiency. This advancement in material science not only enhances the performance of TENGs but also expands their application in more demanding environmental conditions.

**Figure 3:**
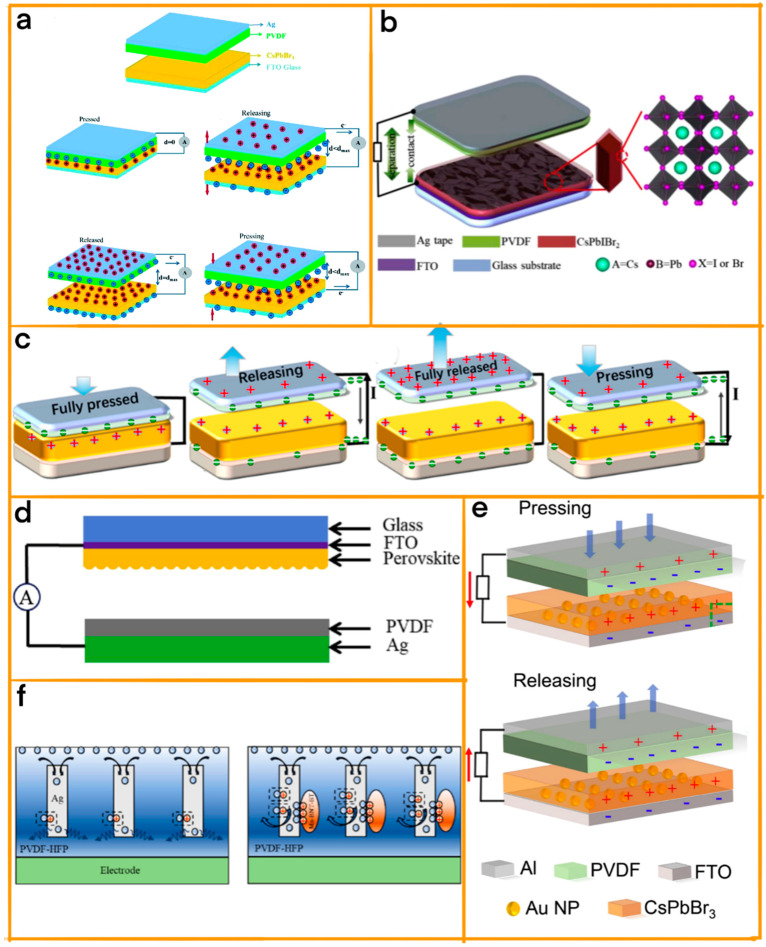
(**a**) The working mechanism of a perovskite TENG. (Royal Society of Chemistry, 2020). (**b**) Illustration of the perovskite TENG device and the crystal structure of inorganic perovskite materials. (Elsevier, 2020). (**c**) Vertical contact-separation mode TENGs with the structure of FTO/perovskite-PVDF/Ag and working mechanism. Electrical output performance. (ACS, 2021). (**d**) Schematic diagram of a perovskite TENG device. (Elsevier, 2020). (**e**) Illustration of the Au/perovskite TENG structure. (Elsevier, 2020). (**f**) Schematic diagram of electron migration in the PVDF-HFP + 5% AgNWs nanofiber tribonegative layer and in the PVDF-HFP + 5% Mn-BNT-BT + 5% AgNWs nanofiber tribonegative layer. (Elsevier, 2022).

In the research conducted by Xie et al. [66], they explored the synergistic effect of combining cesium lead bromide (CsPbBr_3_) perovskite film with nanoplatinum (Pt), achieving a TENG characterized by its unparalleled efficiency and robust output (Figure 3a). This novel integration produced a TENG with an outstanding open-circuit voltage of 273 V and a short-circuit current density of 30.3 mA cm^−2^, both of which are significant improvements over previous models. Moreover, the TENG exhibited an extraordinary instantaneous maximum power density of 1295.5 mW m^−2^. The crux of this exceptional performance lies in the meticulous optimization of the Pt dosage. By doing so, Xie et al. effectively maximized the electron binding energy and enhanced the dielectric properties of the perovskite films.

Building on this advancement, Du et al. introduced a groundbreaking design in TENG technology by incorporating a multilayered Co(OH)(CO_3_)_0.5_/Pt/CsPbIBr_2_ nanoarray (Figure 3b) [67]. The multilayered nanoarray, with its carefully engineered structure, played a crucial role in this enhancement. It not only expanded the contact surface area, allowing for more efficient charge generation, but also fine-tuned the dynamic behavior of the triboelectric charges. This meticulous engineering led to a remarkable improvement in the triboelectric charge density, which was a pivotal factor in the TENG achieving a power density output 4.5 times higher than that of its standard CsPbIBr_2_ counterpart, showcasing the remarkable potential of multilayered nanostructures in enhancing TENG performance.

In a similarly stride, Yu et al.’s research presents a fascinating exploration of the unique properties of halogen-doped inorganic CsPbX_3_ perovskites, specifically focusing on CsPbBr_3_ and CsPbCl_3_ (Figure 3c) [68]. Their study stands out for its insightful use of density functional theory calculations, which were instrumental in examining the surface energy and crystal polarizability of these perovskites. A key discovery of their research was the enhanced polarizability of CsPbCl_3_ in comparison with CsPbBr_3_. This difference in polarizability had a profound impact on the performance of TENGs as it significantly boosted the built-in electric field within the CsPbCl_3_ perovskite materials. Such enhancement led to a greater retention of triboelectric surface charges, culminating in a notably higher power output from TENGs based on CsPbCl_3_.

Similar, Du et al. made substantial strides in advancing TENG technology by leveraging the unique properties of all-inorganic cesium lead halide (CsPbX_3_) perovskites, synthesized through a facile solvent etching method (Figure 3d) [69]. The choice of these perovskites was instrumental in enhancing the TENG’s efficiency, primarily through the augmentation of the triboelectric charge density. The team meticulously investigated how the perovskite films’ electron-donating capacity, influenced by their contact area and dielectric properties, could be optimized to improve TENG performance. Their efforts bore fruit in the form of a CsPbBr_2.6_I_0.4_-based TENG, which demonstrated impressive electrical characteristics, achieving an open-circuit voltage of 192 V and a maximum power density of 1.2 W m^−2^ at a contact frequency of 0.5 Hz.

Then, Wang et al. embarked on an innovative journey to harness the exceptional properties of inorganic CsPbBr_3_ perovskite (Figure 3e) [70]. By adeptly utilizing this material as a dielectric layer, they crafted a highly efficient metal/perovskite Schottky junction, a design choice that proved to be transformative. This junction played a pivotal role in regulating the dynamic behavior of triboelectric charges, adeptly suppressing the charge screen effect which often plagues TENGs. The strategic placement of the perovskite layer facilitated a more efficient electron transfer to the metallic nanoparticles, leading to a significant boost in charge accumulation at the bottom electrode. Such an enhancement in electron dynamics directly translated into an impressive increase in power output, elevating the device’s power density to 3.31 W m^−2^, accompanied by an open-circuit voltage of 240 V and a short-circuit current density of 4.13 μA cm^−2^.

In a similar stride, He et al. adopted an innovative approach by incorporating a blend of conductive silver nanowires (AgNWs) and perovskite oxide Mn-doped (Bi_0.5_Na_0.5_)TiO_3_-BaTiO_3_ (Mn-BNT-BT) nanocrystals within electrospun poly(vinylidene fluoride-co-hexafluoropropylene) (PVDF-HFP) nanofibers (Figure 3f) [71]. This unique composite structure led to an extraordinary increase in power output, a staggering 386% over traditional PVDF-HFP-based nanogenerators. The substantial enhancement in performance can be attributed to a synergistic interplay of factors: the improved dielectric properties owing to the perovskite oxide nanocrystals, the increased capacitance resulting from the Ag NWs, and the enhanced charge trapping capabilities of the composite. Moreover, the enlarged work function of the composite fiber mat played a crucial role, contributing to the overall efficiency of the TENG.

#### 3.1.2. Organic–Inorganic Hybrid Perovskite to Improve the TENG’s Output

Organic–inorganic hybrid perovskites bring together the best of both worlds, combining the flexibility and solution-processability of organic materials with the high charge mobility and stability of inorganic components. Their incorporation into TENGs leads to devices with improved mechanical flexibility, higher power output, and greater efficiency under various operational conditions, particularly in light-responsive applications.

**Figure 4:**
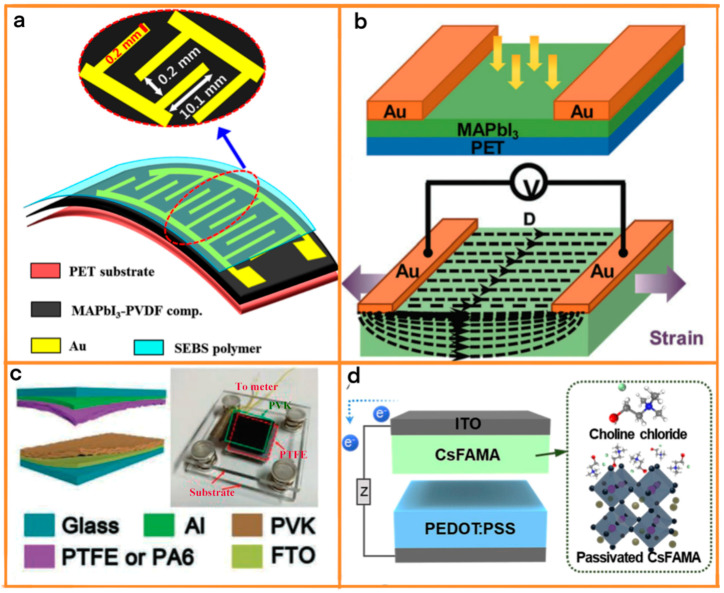
(**a**) Design of a single–structured multifunctional device and characterization of a MAPbI_3_-PVDF composite film: Schematic of multifunctional device. (ACS, 2020). (**b**) Principle of MAPbI_3_ perovskite photodetectors. (Royal Society of Chemistry, 2020). (**c**) Schematic structure and an optical image of the PT–/PA–PVK TENG. (Wiley-VCH, 2020). (**d**) Device structure of PEDOT:PSS–CsFAMA–based DC–TENG and atomic structures of ChCl and CsFAMA. (Elsevier, 2023).

In the innovative realm of IoT applications, Ippili et al.’s research marked a significant milestone by developing a multifunctional device that ingeniously harnesses the combined photoelectric and piezoelectric/triboelectric properties of methylammonium lead iodide-polyvinylidene fluoride (MAPbI_3_-PVDF) composite (Figure 4a) [72]. This device stands out for its extraordinary versatility, adeptly functioning as both an energy harvester and a sensor. In its role as a harvester, it showcases notable light-driven outputs in both piezoelectric and triboelectric modes, with its performance exhibiting remarkable variability under different illumination conditions. The device further extends its capabilities by acting as a self-powered photodetector, demonstrating outstanding responsivity. Additionally, it doubles as a highly sensitive pressure sensor, exhibiting exceptional sensitivity and robust mechanical durability. The study diligently highlighted the pivotal role of light in influencing the device’s output across varying pressures and illumination conditions, revealing significant enhancements in output power density, a remarkable attribute attributable to the photoactive nature of MAPbI_3_.

Expanding the horizons of photodetector technology, Nie et al. introduced a groundbreaking device based on polycrystalline MAPbI_3_ (Figure 4b) [73]. This study brought to light the numerous advantages of polycrystalline materials, notably their ease of fabrication, cost-effectiveness, and high conversion efficiency, offering a pragmatic alternative to single crystals. The performance of this device was notably augmented by the piezophotoelectronic effect, which not only enhanced its piezoelectric performance but also allowed for meticulous fine-tuning of this aspect. The research delved deep into the workings of strain-controlled Schottky barriers in MAPbI_3_ devices, revealing how controlled strain can significantly alter the distribution of polarized electric fields. This alteration plays a crucial role in enhancing electron–hole pair separation and reducing recombination, key factors in the efficiency of photodetectors. The insights gleaned from this study pave the way for a myriad of applications, particularly in sensor networks and other advanced technological fields, heralding a new era in the integration of polycrystalline perovskite materials in cutting-edge electronic devices.

Building on the theme of material innovation in TENG technology, Huang et al.’s study presented a groundbreaking approach that significantly enhanced TENG performance through the use of organic–inorganic hybrid perovskite materials [74]. Their research was centered on the idea of manipulating the chemical composition of perovskite films and inducing ion migration, which effectively led to substantial changes in conductivity type, surface potentials, and electron affinities (Figure 4c). In an exemplary display of material adaptability, their TENGs, when paired with either PTFE or nylon, showcased the ability to function as either positive or negative tribomaterials. This level of adaptability, achieved through a harmonious combination of optimized chemical composition ratios and electric field-induced ion migration, was instrumental in driving a considerable increase in output voltage and power density. Huang et al.’s work not only set a new benchmark in TENG material design but also paved the way for more refined strategies in enhancing TENG performance, bridging the gap between theoretical research and practical energy-harvesting solutions.

Seamlessly transitioning from Huang et al.’s advancements, Lee et al.’s comprehensive study further pushed the boundaries of TENG technology by harnessing the potential of perovskite materials [75]. Their pioneering work involved the design of a direct current-generating TENG (DC-TENG), leveraging a sophisticated tribovoltaic p-n junction composed of n-type Cs_0.175_FA_0.750_MA_0.075_Pb(I_0.880_Br_0.120_)_3_ (CsFAMA) and p-type PEDOT:PSS. This innovative approach yielded a remarkable initial DC power output, which saw a significant enhancement following the strategic incorporation of choline chloride (ChCl) (Figure 4d). The ChCl served a crucial role in passivating internal defects within CsFAMA, thereby leading to substantial improvements in the triboelectric charge density, carrier mobility, and built-in potential. This research not only underscored the effectiveness of chemical treatment in augmenting the properties of perovskite materials but also established new paradigms in the development of high-performance DC-TENGs. Lee et al.’s findings effectively demonstrate how strategic material engineering and chemical interventions can result in significant advancements in TENG technology, forming a cohesive narrative with Huang et al.’s study in the continuous pursuit of innovation in energy-harvesting materials. Table 1 summarized the performance of several perovskite-based TENGs, including the materials used as friction layers and the open-circuit voltage, current density, and power density.

### 3.2. Optical Coupling Effect of Perovskite-Based TENGs

Leveraging the optical properties of perovskites, TENGs can be optimized through optical coupling, a technique that enhances electrical output by harnessing both mechanical and light energies. This dual-energy-harvesting approach not only increases the power output of TENGs but also broadens their application scope in areas such as wearable technology and integrated self-powered systems. The synergy between the triboelectric effect and photoelectric conversion in perovskites paves the way for next-generation energy-harvesting devices.

**Figure 5:**
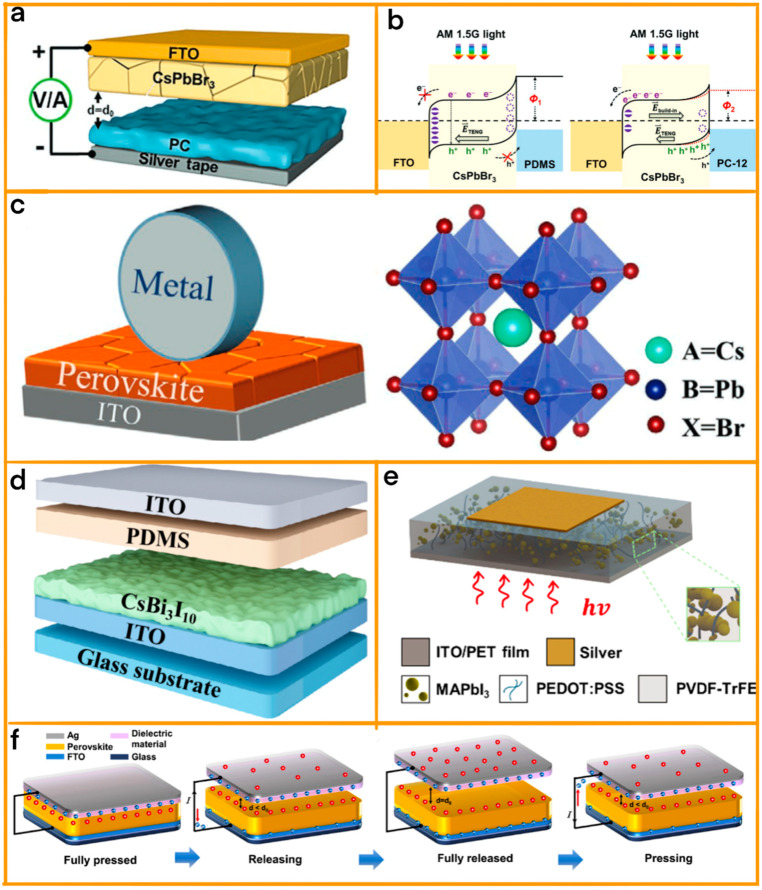
(**a**) Schematic diagram of the hybrid TENG device and energy level alignment diagrams of the hybrid TENGs with pristine PDMS and (**b**) PC composite film under friction-illumination working condition. (Wiley-VCH, 2021). (**c**) Schematic structure of a rolling–mode DCTENG based on metal/perovskite Schottky junction and crystal structure. (Wiley-VCH, 2022). (**d**) The structure and characterization of a TENG: the schematic structure of the CBI/PDMS TENG. (Elsevier, 2023). (**e**) Schematic image of a solar cell structure fabricated with the composite thin films. (Elsevier, 2023). (**f**) Schematic diagram of the vertical contact–separation CsPb_1−x_M_x_Br_3_ perovskite TENG and the working mechanism of a full “press–release” cycle. (Elsevier, 2020).

Guo et al. orchestrated a significant leap forward with their development of a TENG that effectively harnessed the properties of cesium lead bromide (CsPbBr_3_) halide perovskite (Figure 5a,b) [76]. This material played a crucial role in their design, functioning as the primary friction layer that enabled the TENG to exploit a synergistic triboelectric–photovoltaic effect. This innovative approach allowed for the efficient collection of photogenerated charge carriers, thereby substantially enhancing the short-circuit current output of the TENGs. A salient aspect of their design was the integration of a hole-extraction polydimethylsiloxane (PDMS)-multiwall carbon nanotube (MWCNT) composite film, serving dual purposes as a negative triboelectrification layer and as a collector of photogenerated holes. Operating in a vertical contact-separation mode, this hybrid TENG was meticulously engineered, with precise adjustments in MWCNT loadings leading to a remarkable increase in charge quantity due to the triboelectric–photovoltaic coupling effect. The device had the capability to achieve a transferred charge of 0.358 mC under illumination, a figure nearly 30,000 times higher than that of a standard PDMS-based TENG.

In another development, Hu et al. introduced a direct-current triboelectric nanogenerator (DC-TENG) utilizing the unique properties of all-inorganic CsPbBr_3_ perovskite (Figure 5c) [77]. Their approach revolutionized the conversion of mechanical energy into direct current electricity, concurrently harnessing solar energy. Distinct in its rolling-mode design, which diverged from the conventional sliding-structure TENGs, the device incorporated a dynamic Al/CsPbBr_3_ Schottky junction. This key innovation significantly amplified the device’s efficiency under AM 1.5 light illumination, culminating in an impressive output voltage of 3.69 V and a current density of 11.46 Am^−2^—a 4.7-fold increase over its performance in dark conditions.

In the pursuit of renewable energy technologies, Wei et al. made a pioneering contribution by incorporating lead-free bismuth halide perovskite CsBi3I10 (CBI) into their TENG design (Figure 5d) [78]. This innovative integration marked a notable advancement in TENG technology, with CBI being used for the first time as a triboelectric material. The CBI-based TENG showcased exceptional performance, achieving a high voltage of 158 V and a current density of 45.5 mA m^2^ in dark conditions. Remarkably, these performance metrics were further enhanced under illumination, thanks to the triboelectric–photoelectric coupling effect.

Then, Kim et al.’s study took a significant leap forward in TENG technology, focusing on the integral role of perovskite materials in elevating device performance [79]. Their research led to the development of a composite layer that ingeniously combined PEDOT:PSS, PVDF-TrFE, and methylammonium lead triiodide (MAPbI_3_) particles (Figure 5e). This strategic composition was thoughtfully designed to harness the distinctive properties of each component, resulting in a substantial enhancement in the output current density, particularly under light conditions. The inclusion of MAPbI_3_ particles played a pivotal role in this breakthrough. These particles, known for their ability to efficiently generate electron–hole pairs when exposed to light, significantly amplified the TENG’s output current. Furthermore, the encapsulation of MAPbI_3_ within PVDF-TrFE was a critical step in addressing degradation issues commonly associated with perovskite materials, thereby greatly improving the stability of the composite layer.

Finally, Wang et al.’s comprehensive study advanced our understanding of the triboelectric properties of perovskite materials, particularly focusing on cesium lead tri-bromine (CsPbBr_3_) and its variants doped with alkaline earth ions (Figure 5f) [80]. They integrated these materials into vertical contact-separation TENGs and systematically explored their triboelectric charging affinities and polarities. This exploration included detailed analysis of the electrical output performance and dielectric properties, along with evaluations of work function, bond energy, and electron binding energy. The study provided a refined triboelectric series with these perovskite materials, offering quantified insights into their triboelectric charging polarity. The research delved into three distinct configurations of TENGs—dielectric/perovskite, carbon/perovskite, and perovskite solar cells—under both dark conditions and illumination. A key finding was the role of photoinduced charges, which not only enhanced triboelectrification charges but also impacted the charging polarity of perovskites, offering new perspectives on their behavior under varying conditions.

### 3.3. High Stability of Perovskite-Based TENGs

**Figure 6:**
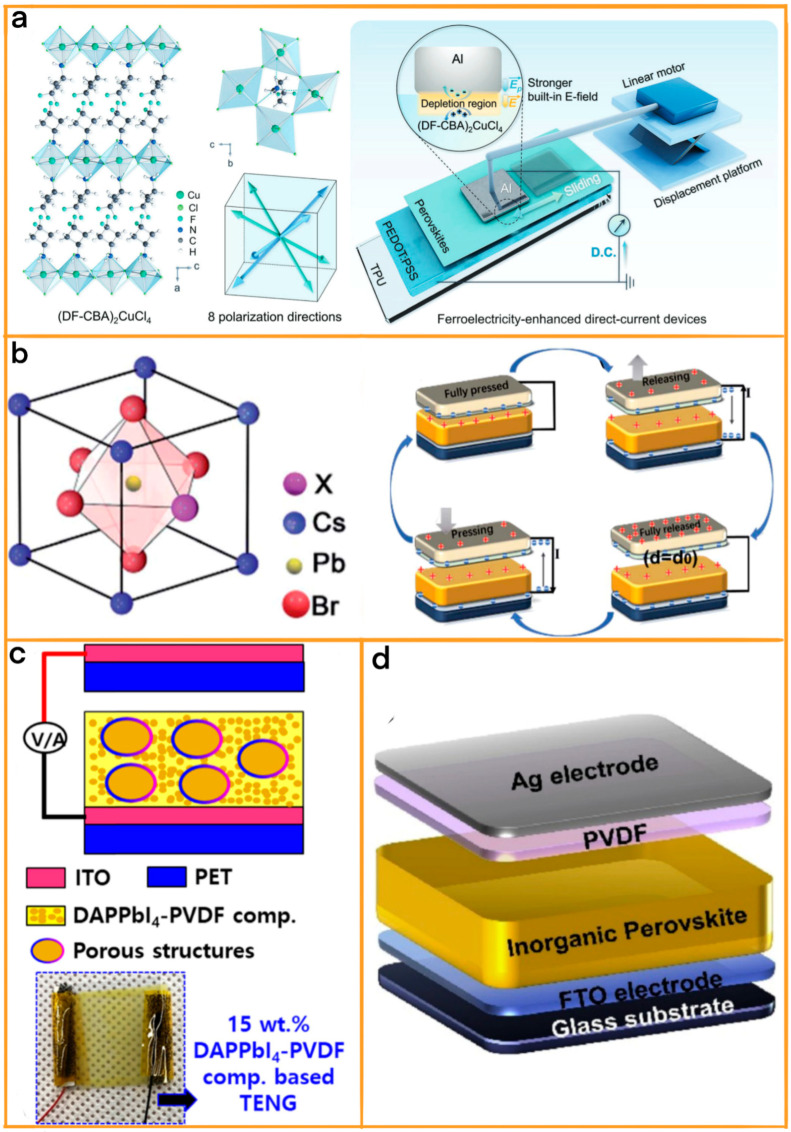
(**a**) Demonstration of the materials, device structures, experimental setup, and preparation process of a (DF-CBA)_2_CuCl_4_-based TVNG device, including side and top views of the crystal structures and eight polarization directions. (Wiley-VCH, 2021). (**b**) The crystal structure of an inorganic halide perovskite unit and an illustration of charge generation, distribution, and transfer in a press–release cycle. (Royal Society of Chemistry, 2020). (**c**) Schematic illustration of a fabricated DAPPbI_4_–PVDF TENG and a photograph of the device. (Elsevier, 2022). (**d**) Schematic diagram of the “dielectric-on-perovskite” TENG with vertical contact-separation mode. (Elsevier, 2020).

TENGs have seen substantial advancements through the integration of perovskite materials, known for their remarkable electrical and physical properties. In this context, Peng et al.’s pioneering work in developing a tribovoltaic nanogenerator (TVNG) marks a significant leap in semiconductor energy technology [34]. Their innovation, a dynamic Al/(DF-CBA)_2_CuCl_4_ Schottky junction TVNG, harnesses the unique properties of lead-free perovskite material ((DF-CBA)_2_CuCl_4_) (Figure 6a). Central to its functionality is the perovskite’s exceptional multiaxial ferroelectricity, enabling substantial modulation of surface charge and work function. This modulation results in a significantly increased work function difference with Al electrodes, which is vital for creating a stronger built-in electric field at the Schottky interface, superior to traditional semiconductors and halide perovskites. This enhanced electric field contributes to a more than 15-fold increase in current output, showcasing the device’s exceptional flexibility and durability. Remarkably, it maintains stable performance over 20,000 cycles and can withstand extreme mechanical deformations, demonstrating the substantial stability improvements that perovskite materials bring to TENG technology.

Further exploring the realm of perovskite-enhanced TENGs, Yu et al. delved into the triboelectric properties of all-inorganic CsPbX_3_ halide perovskites in vertical contact-separation TENGs. Their comprehensive study offers crucial insights into the role of halide ions (Cl^−^, Br^−^, and I^−^) in tailoring the triboelectric behavior of perovskite materials (Figure 6b) [81]. By meticulously investigating the perovskites’ triboelectric charging behaviors and focusing on parameters such as power density, charging capacity, and figure of merit, they identified CsPbCl_3_ perovskite TENGs as standout performers. These TENGs achieved a remarkable maximum power output of 3.04 W m^−2^ and demonstrated extraordinary long-term stability, maintaining consistent performance over 60 days in an ambient atmosphere.

Then, Ippili et al. introduced a groundbreaking TENG leveraging the unique attributes of two-dimensional layered halide perovskites, particularly DAPPbI_4_ blended with PVDF (Figure 6c) [82]. This innovation led to the creation of a DAPPbI_4_–PVDF composite TENG, a device that stood out for its extraordinary environmental stability and markedly enhanced piezoelectric/ferroelectric properties, setting it apart from traditional 3D halide perovskites (3D-HPs). The innovative composite, containing 15 wt% DAPPbI_4_, significantly improved the electro-active β-phase of PVDF. This advancement translated into a high dielectric constant and a robust piezoelectric coefficient, culminating in a TENG that delivered exceptional output voltage and current density, along with an unprecedented power density. Notably, the TENG also exhibited superior pressure sensitivity, a feature crucial for diverse applications.

Finally, Wang et al.’s study explored the untapped potential of all-inorganic cesium lead tri-bromide (CsPbBr_3_) perovskites, a material traditionally known for its optical features, in the realm of TENGs [83]. This significant leap in the field of optoelectronic and electronic nanodevices was achieved through an innovative approach that involved doping the CsPbBr_3_ lattice with barium ions (Ba^2+^) (Figure 6d). This process resulted in the creation of modified CsPb_1−x_BaxBr_3_ perovskites, a strategic alteration that significantly enhanced the material’s dielectric constant, grain size, and surface contact area. The resultant optimization of the CsPbBr_3_-based TENG was nothing short of remarkable, delivering an open-circuit voltage of approximately 220 V and a peak power density of 3.07 W m^2^. Such impressive performance enabled the device to power over 80 LED lights, a testament to its impressive energy conversion capabilities. Moreover, the TENG displayed exceptional durability and stability, maintaining consistent performance over extended periods, even under varying environmental conditions.

## 4. Hybrid Energy Collection System

The hybrid energy collection system represents a progressive step in sustainable energy technologies, combining various energy-harvesting mechanisms to optimize efficiency and reliability. This system typically integrates TENGs, solar panels, and sometimes wind energy harvesters to collect energy from multiple environmental sources. Such systems are pivotal in addressing the growing demand for renewable energy sources and mitigating the intermittency issues associated with traditional renewable energy systems like solar and wind alone. By diversifying the energy collection methods, hybrid systems can provide a more consistent and reliable power supply, essential for both grid-connected and off-grid applications.

### 4.1. TENG–Solar System

The TENG–solar system merges the capabilities of TENGs with solar energy technology. TENGs, which convert mechanical energy into electricity based on triboelectric effects and electrostatic induction, are combined with photovoltaic cells that convert solar energy into electricity. This integration capitalizes on the complementary nature of these technologies: TENGs can harness energy from environmental movements and human activities, while solar cells exploit sunlight. The combination leads to a more robust and versatile energy-harvesting system, capable of functioning under various weather conditions and in different environments, making it highly suitable for sustainable and autonomous power generation.

**Figure 7:**
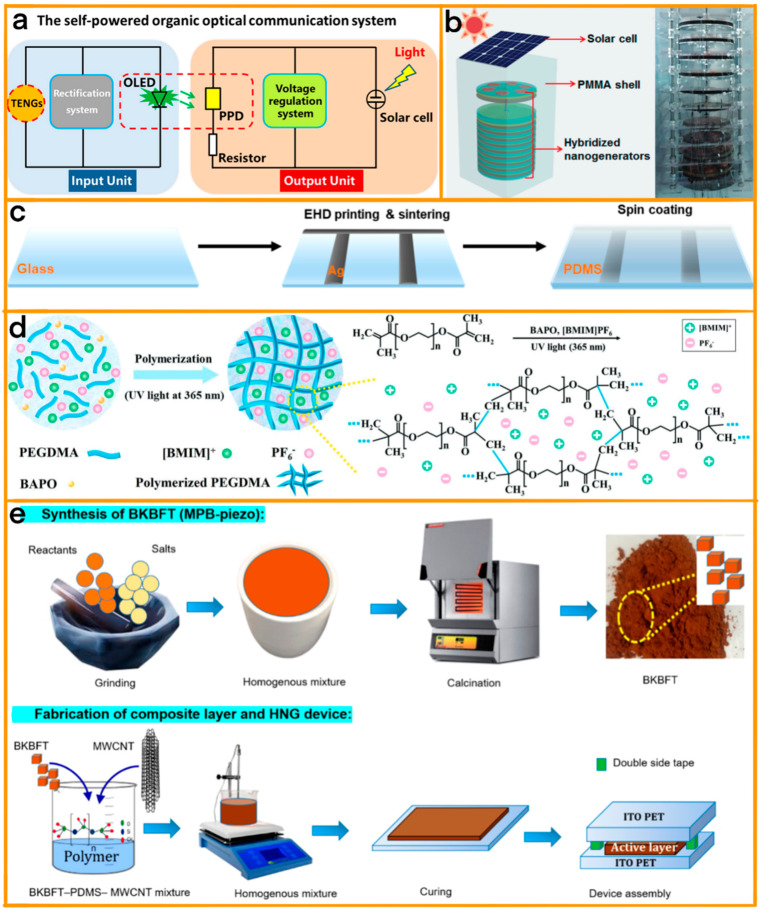
(**a**) Schematic diagram and photographs of a self-powered optical communication system (SOCS). (Elsevier, 2019). (**b**) Structure and working principle of the hybridized electromagnetic-TENG. (Springer, 2022). (**c**) Schematic diagram of the PA–TENG fabrication process. (Elsevier, 2022). (**d**) Schematic illustration of photopolymerization of PEGDMA with bond breaking and forming details. (Wiley-VCH, 2023). (**e**) Schematic illustrations of the synthesis of BKBFT via the molten salt method and the preparation and fabrication of the composite film and HNG devices. (ACS, 2023).

In the innovative realm of hybrid energy collection, Guo et al.’s work stands out with their self-powered organic optical communication system (SOCS) (Figure 7a) [84]. This novel system ingeniously integrates perovskite photodetectors (PPDs) with an organic light-emitting diode (OLED) driven by TENGs and a solar cell-powered PPD. The SOCS can convert mechanical signals into light and then into voltage signals, enabling data transfer between electrically insulated units. A remarkable capability of this system is encoding mechanical tapping on TENGs as binary data, facilitating substantial data transmission and even controlling a robotic hand, demonstrating advanced human–machine interaction. The system’s adaptability is highlighted by its ability to handle a voltage difference of up to 2000 V between isolated units, showcasing its applicability in extreme environments.

Cheng et al.’s study contributes to this field with a hybridized energy harvester designed for efficient energy collection and wireless power transmission to mobile electronic devices [85]. This device combines electromagnetic–TENG units with a flexible solar cell, efficiently harvesting energy from various sources at a rotational speed of 500 rpm (Figure 7b). The electromagnetic–TENGs yield a significant output current of approximately 630 mA, capable of wireless transmission over a distance of about 100 cm, enabling real-time charging of devices like mobile phones and hygrometers. The harvester consists of ten electromagnetic–triboelectric units, each producing peak output powers of 65.7 mW for the EMG and 429 mW for the TENG.

#### 4.1.1. Improving the Energy Collection Efficiency

Improving the energy efficiency of TENG–solar systems involves optimizing the integration of mechanical and solar energy harvesting to maximize power output and minimize losses. Research in this area focuses on enhancing the materials, structures, and interfaces used in these systems to improve their energy conversion rates and operational stability. This may include the development of more efficient TENG materials, advanced solar cell technologies like perovskite solar cells, and innovative designs that effectively combine these technologies. Improving energy efficiency in TENG–solar systems is crucial for their broader adoption in applications ranging from wearable electronics to smart cities, where they can provide a sustainable and independent power source.

In the pursuit of enhancing the energy efficiency of TENG–solar systems, Im et al.’s study introduced a significant innovation with the transparent water–solid contact triboelectric nanogenerator (PA-TENG) integrated with a solar cell (Figure 7c) [86]. Specifically designed for regions with pronounced wet and dry seasons, especially in tropical climates, PA-TENG featured a highly transparent and conductive electrohydrodynamic jet-printed Ag nanoparticle (Ag NP) electrode. This electrode demonstrated superior performance in comparison with the conventional indium tin oxide (ITO) electrode used in ITO-TENG. The PA-TENG achieved a maximum power output of 1.17 W m^−2^ and showcased impressive optical transmittance, with values peaking at 96% and averaging 91%. This high transmittance and power output were attributed to the effective charge-inducing ability at the Ag NP/polydimethylsiloxane (PDMS) interface and the low sheet resistance of the printed Ag NP electrode. In contrast, the ITO-TENG’s power output was significantly lower, and its integration with a solar cell resulted in a greater reduction in power density compared with the PA-TENG. The superior performance of the PA-TENG was further highlighted by its ability to maintain higher transparency and power output during both rainy and sunny days, making it more suitable for integration with solar cells than the ITO-TENG. This integration only marginally reduced the PA-TENG’s power density by 3.6% compared with a 5.2% reduction for the ITO-TENG.

Wu et al. presented advancements in TENGs utilizing ionogel-based technology (I-TENGs), highlighting a novel approach to enhance energy-harvesting capabilities [87]. The study primarily focuses on the intricate relationship between the structure and properties of ionogels and their effects on TENG performance (Figure 7d). It is shown that the conductivity of ionogels, composed of a crosslinking monomer and an ionic liquid, is significantly influenced by the cross-link density, impacting the efficiency of charge channels within the ionogel. This understanding is pivotal in optimizing the ionogel formulation, where an appropriate ratio of cross-linker to ionic liquid is essential for forming effective charge channels, although an excess of ionic liquid proves counterproductive. The research further explores the application of these optimized ionogels in I-TENGs for harvesting energy from water droplets, correlating their structural properties to performance efficiency. A notable breakthrough in this research is the enhancement of I-TENG performance through the introduction of surface polymer brushes synthesized via a universal vapor-phase polymerization process. These brushes confer a slippery surface to the I-TENGs, facilitating quicker water droplet movement and thus increasing energy generation. Additionally, these polymer brushes provide antireflective properties, making them suitable as a surface covering for solar cells, thereby boosting solar cell performance.

Finally, Pranab et al.’s study introduced a flexible hybrid nanogenerator (HNG) leveraging the unique properties of perovskite materials to achieve high-output performance and flexibility (Figure 7e) [88]. This HNG, comprising a composite of lead-free piezoelectric ceramic oxide (Bi_0.785_K_0.035_Ba_0.180_) (Fe_0.750_Ti_0250_) O_3_ known as BKBFT/MPB-piezo and the triboelectric polymer poly(dimethylsiloxane) (PDMS), demonstrates remarkable improvements in piezoelectric properties at the morphotropic phase boundary (MPB). The addition of 1 wt% multiwalled carbon nanotubes (MWCNTs) to the 89 wt% PDMS and 10 wt% MPB-piezo composites further boosts the device’s efficiency by forming nanoelectrical bridges that facilitate charge transfer and improve structural homogeneity. The resulting HNG device, named HNGC10-1, exhibits a peak-to-peak open-circuit voltage of 22 V, a short-circuit current of 1.8 μA, and a power density of 72 nW cm^−2^, functioning effectively without the need for electric field poling.

#### 4.1.2. Expanding the Energy Collection Channels (Using Raindrop Energy)

The TENG–solar system for raindrop energy collection represents an innovative approach to all-weather energy harvesting. It incorporates the traditional solar energy collection with the added capability of harvesting energy from raindrops using TENG technology. This system ensures continuous energy generation as TENGs can generate electricity from the impact of raindrops when solar panels are less effective under cloudy and rainy conditions. The dual-functionality of these systems makes them highly suitable for regions with frequent rain, providing a more consistent and reliable energy source compared with standalone solar systems.

**Figure 8:**
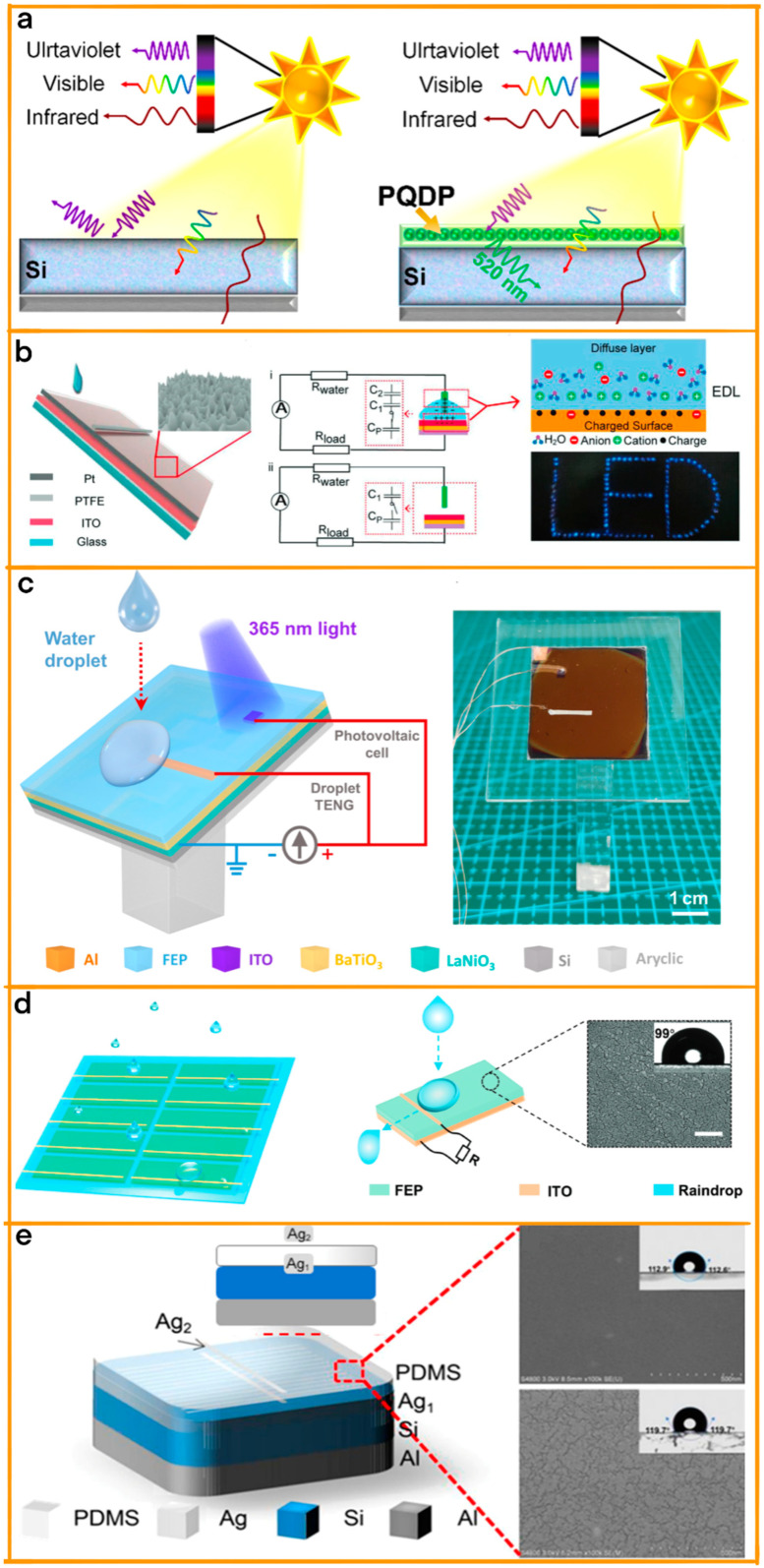
(**a**) Schematic diagram of the sunlight response to the pristine Si solar cell and the down–conversion mechanism of the PQDP–based solar cell. (Elsevier, 2023). (**b**) Physical structure and electrical outputs of I–TENG, including the structural diagram, the equivalent working circuit diagram, the electric–double–layer model, and a photograph of the I–TENG lighting up LEDs. (Wiley-VCH). (**c**) Schematic diagram of the hybridized nanogenerator and optical image of the device. (Elsevier, 2023). (**d**) Basic principle to design the R–TENG array, including the schematic diagram, the structure diagram of a single unit, an SEM image, and a contact angle photograph of the FEP film. (Wiley-VCH). (**e**) Schematic diagram for a TENG/Si tandem hybrid solar cell and top–view SEM images. (Elsevier, 2021).

Yuan et al.’s significant contribution to the field of hybrid power generation systems is marked by their innovative tandem hybrid solar cell, a system ingeniously integrating a TENG with a crystalline silicon (Si) solar cell (Figure 8a) [89]. This integration artfully harnesses both solar and rain energy, utilizing a top-cell layer composed of a perovskite quantum dots-embedded polydimethylsiloxane (PQDP) film. The PQDP film serves a twofold purpose: as a triboelectric charge enhancement layer for capturing energy from raindrops and as a down-conversion layer, transforming ultraviolet light into wavelengths absorbable by the Si cell. This results in a notable increase in the solar cell’s power conversion efficiency (PCE), from 18.47% to 20.16%, attributable primarily to the antireflection properties of the PQDP film and the enhanced down-conversion ability of the CsPbBr_3_ quantum dots doped with Ce^3+^ ions.

In a complementary advancement, Zheng et al. introduced a novel quantitative analysis method known as the kinetic energy calculation and current integration (KECCI), which revolutionizes the understanding of the mechanical-to-electrical energy conversion process in TENGs (Figure 8b) [90]. This breakthrough was achieved through the development of a high-performance TENG exhibiting impressive specifications, including a 1.25 mA short-circuit current, 150 V open-circuit voltage, and a remarkable 24.89% energy conversion efficiency. This efficiency was attained by optimizing biomimetic surface structures and implementing an innovative instant switch design. Zheng et al.’s study goes a step further by proposing a multilayered TENG design.

Then, Bao et al. made a significant contribution with their design of a groundbreaking hybridized nanogenerator [91]. This device ingeniously integrates a photovoltaic cell (PVC) with a TENG, enabling the simultaneous scavenging of energy from both light and liquid droplets (Figure 8c). The unique feature of this design is the shared bottom electrode between the PVC and the TENG, which plays a crucial role in enhancing the energy-harvesting efficiency of the system. This hybridized nanogenerator is capable of generating peak currents of 16.96 μA and peak voltages of 0.83 V, achieving a maximum peak power density of 3.71 mW m^−2^ and an output energy density of 15.36 mJ m^−2^. Notably, the TENG’s pulse–like output primarily determines the maximum hybridized peak power, while the continuous output from the PVC significantly influences the maximum hybridized output energy. Furthermore, the inclusion of a BaTiO_3_ (BTO) film in the TENG component enhances the retention of surface charges, thereby improving the energy capture efficiency from liquid droplets.

Ye et al.’s R–TENG array represents a significant advancement in energy harvesting from irregular raindrops, efficiently capturing energy from natural sporadic rainfalls with its effective electrode structure (Figure 8d) [92]. In comparison with traditional solar cells, the R–TENG shows superior performance, achieving an average power density of 40.80 mW m^−2^ in rainy conditions. The integration of this R–TENG array with solar panels, as envisaged by Bao et al., demonstrates its potential for consistent energy output in varying weather conditions. This integration aligns with the innovative approaches of Yuan et al. and Zheng et al., who focused on harnessing solar and rain energy and enhancing energy conversion efficiency, respectively.

Finally, Zhao et al.’s pioneering work in the development of the TENG/Si tandem hybrid solar cell is a testament to the evolving landscape of hybrid energy collection systems (Figure 8e) [93]. Their design is a striking example of innovation as it uniquely harnesses energy from both solar and rain sources. By integrating a silver/polydimethylsiloxane (Ag/PDMS) top subcell with a monocrystalline Si solar cell, the device achieves the capability of simultaneous energy capture from diverse environmental sources. This groundbreaking hybrid cell demonstrates a peak power conversion efficiency (PCE) of 22.04% under standard solar illumination, along with a remarkable maximum power output of 147 μW under raindrop stimuli.

#### 4.1.3. Improve the System Stability

Enhancing the stability of TENG–solar systems is crucial for their long-term functionality and reliability. Research in this area focuses on improving the durability and resilience of TENGs and solar cells, especially in harsh environmental conditions. This includes developing materials that can withstand environmental stressors such as moisture, temperature fluctuations, and mechanical wear and tear. Additionally, innovations in device design, such as incorporating self-healing materials and protective coatings, play a significant role in extending the lifespan and maintaining the efficiency of these hybrid systems. Stability is a key consideration for the widespread adoption of TENG–solar systems in various applications, from industrial to residential settings.

**Figure 9:**
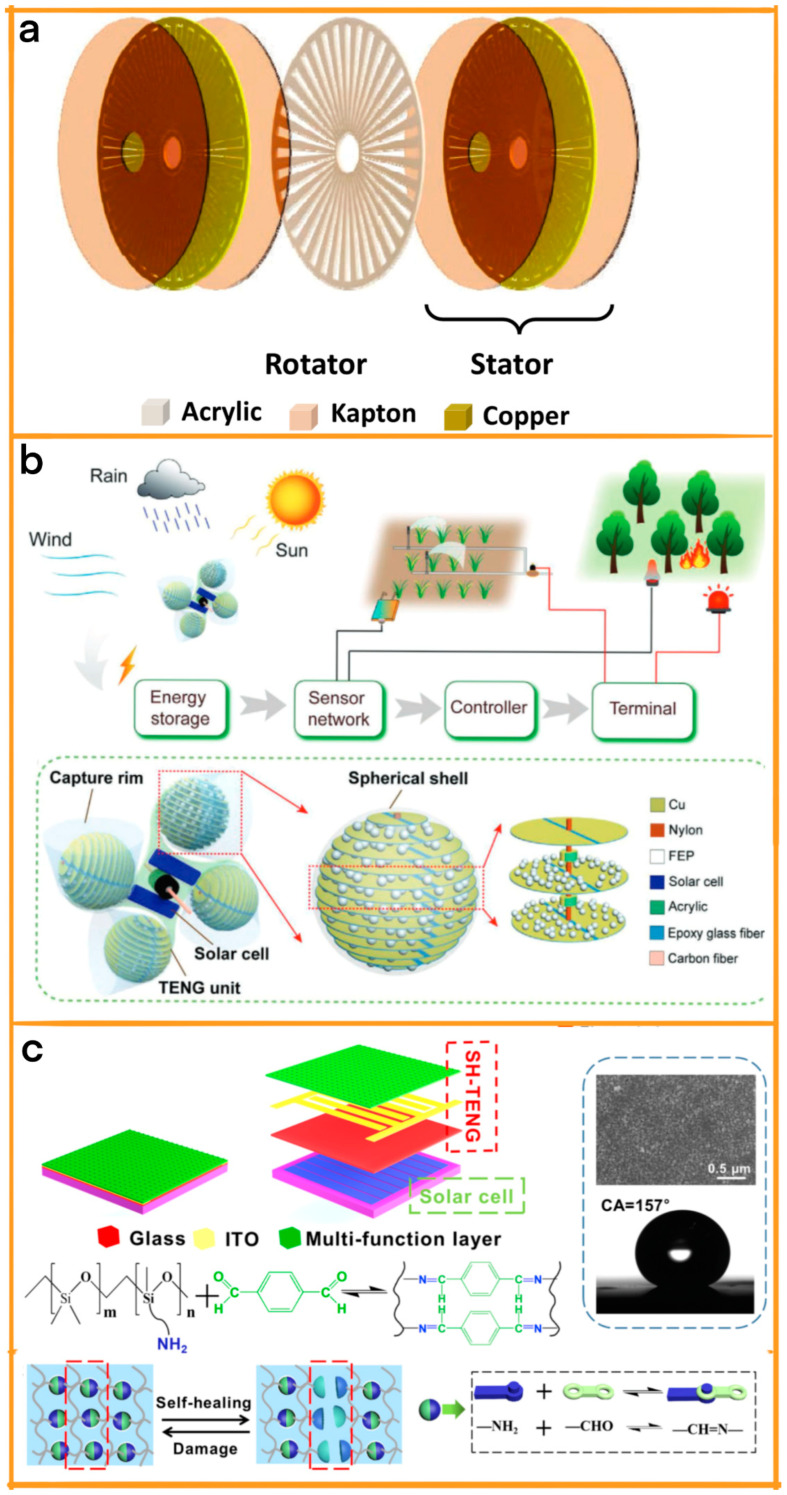
(**a**) Characterization of the multilayered disk TENG performance and material composition. (Elsevier, 2020). (**b**) Concept, structure, and working principle of the hybrid all-in-one power source (AoPS) for harvesting environmental energy. (Wiley-VCH). (**c**) Schematic diagram of the hybrid energy system with the SH-TENG and solar cell, mechanism of self-healing SH-PDMS, chemical structures, SEM image, and contact angle image. (Elsevier, 2021).

In the quest to enhance the stability of TENG-assisted solar systems in extreme environments, Lin et al.’s research stands out. They introduced a groundbreaking approach to anodic bonding by incorporating a TENG as the power source (Figure 9a) [94]. This TENG-based anodic bonding system is notable for its low current and minimal charge transfer requirements, eliminating the need for pretensile strength in bonded pairs. Remarkably, the system can tightly bond a silicon–glass interface of 100 mm^2^ in just 40 s, showcasing efficiency comparable to commercial power sources. A unique feature of this system is its use of the pulse current signal from a multilayered disk TENG, allowing for controlled bonding by adjusting the TENG’s rotation rate. This method results in lower bonding current, fewer required charges, and robust tensile strength of bonded pairs, supporting the trend toward more integrated and complex MEMS devices. The study demonstrates that even with the TENG’s low current output, the bonding performance remains highly efficient. For example, while a traditional DC power source needs 40 mC to achieve complete bonding, the TENG-driven system requires only 7.8 mC for a similar bonding area. Furthermore, this approach achieves a maximum bonding strength of 15.38 MPa for silicon–glass pairs at a TENG rotation speed of 600 rpm, with a bonding time of around 30 s for 100 mm^2^. The system also successfully bonds triple-layered structures like glass–silicon–glass in a two-step process, reaching a maximum bonding strength of 8.49 MPa.

Meanwhile, Xu et al. addressed the need for versatile and robust energy-harvesting technologies in the Internet of Things (IoT) domain, where electronic devices are increasingly integrated into various environments [95]. They pioneered a hybrid all-in-one power source (AoPS) adept at harvesting energy from diverse environmental sources. This novel device effectively combines high-performance spherical TENGs with solar cells, creating a system that can harness energy from wind, rain, and sunlight (Figure 9b). The AoPS’s unique design, featuring spherical TENG units with a robust packaged structure, enables efficient energy collection from fluid movements, ensuring nearly continuous direct current output and a significant average power output of 5.63 mW from just four TENG units. This is further enhanced by the integration of solar cells, offering a complementary energy source. The device’s practicality extends to various applications including self-powered soil moisture control, forest fire prevention, and pipeline monitoring, demonstrating its adaptability and reliability in harsh environments.

Finally, Yang et al. designed a novel hybrid energy system that integrates a solar cell with a self-healing/self-cleaning TENG to efficiently harvest energy from both sunlight and raindrops (Figure 9c) [96]. This system addresses the limitations of solar cells in adverse weather conditions and reduces potential mechanical damage. The unique TENG, composed of conductive indium tin oxide (ITO) electrodes and a multifunctional silicone elastomer layer, exhibits superhydrophobicity and self-healing properties. This layer, combining fluorinated nanosilica organosilane composite resin (OSCR) and self-healing polydimethylsiloxane elastomer (SH-PDMS), is capable of 100% healing efficiency at ambient conditions. The SH-TENG generates a peak voltage of 6 V and a current of 0.8 μA, maintaining performance even after surface damage, which self-heals within 0.5 h at room temperature. This hybrid system not only provides a protective layer for the solar cell, enhancing its durability, but also maintains high energy-harvesting efficiency due to the SH-TENG’s transparency. Table 2 summarizes the achievements and applications of a perovskite-based hybrid energy-collecting system, which basically consists of a TENG structure (structure A) and a solar cell structure (structure B). After the combination, the energy sources are expanded, and the output performance of the TNEG and the PCE of the solar cell are improved.

### 4.2. TENG–Solar–Wind System

The TENG–solar–wind system is a comprehensive energy-harvesting solution that combines the principles of triboelectric, photovoltaic, and wind energy conversion. This system is designed to capture energy from multiple natural sources: solar radiation, wind, and mechanical motions (including raindrops and ambient vibrations). The integration of these diverse energy-harvesting methods ensures a more consistent and reliable energy supply, making the system particularly advantageous for areas with variable weather patterns. This multifaceted approach not only enhances the overall efficiency and output of the energy-harvesting system but also contributes to a more sustainable and eco-friendly power generation model, aligning with global efforts to transition toward renewable energy sources.

**Figure 10:**
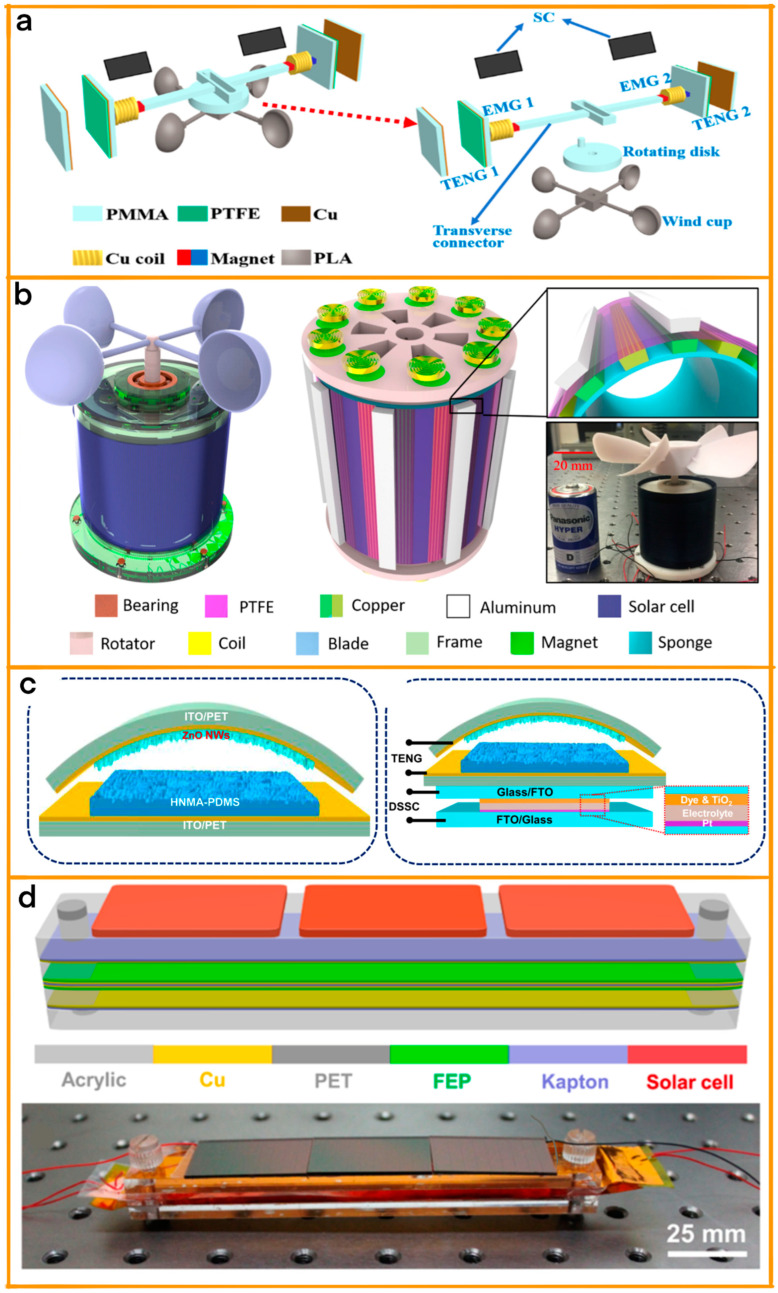
(**a**) Architecture of the HEHD and schematic illustration of the local view, consisting of TENGs, EMGs, and commercial SCs. (Elsevier, 2022). (**b**) Structural design of the WH-EH for wireless natural disaster monitoring, including functional components, detailed structure of the TENG unit, and an optical image of the device. (Elsevier, 2018). (**c**) The arch-shaped TENG integrated with HNMA-PDMS and ZnO NWs and the hybrid energy cell consisting of a TENG and a DSSC. (ACS, 2016). (**d**) Schematic diagram and photograph of the fabricated hybridized nanogenerator. (ACS, 2016).

Wang et al. have made a notable advancement in smart agriculture with their hybridized energy-harvesting device (HEHD), which expertly harnesses both wind and solar energy [97]. This innovative device integrates two electromagnetic generators (EMGs), two TENGs, and two solar cells (SCs), thereby enabling efficient collection of environmental energy (Figure 10a). A key feature of the HEHD is its ability to convert rotational motion into translational motion through a transverse connector, enhancing the durability of the TENGs and optimizing energy generation. The device demonstrates impressive output performance, with the TENGs and EMGs increasing their efficiency as wind speeds rise, reaching an open-circuit voltage of nearly 570 V and a short-circuit current of 40 μA and 0.8 V and 1.85 mA at a wind speed of 7 ms^−1^, respectively. The system also includes a self-powered temperature and humidity monitoring setup, which incorporates a custom mobile application and a commercial sensor for remote monitoring and multipoint connection of environmental data. This system is complemented by a farm peripheral security alarm system, also based on the HEHD, which enables passive remote infrared monitoring.

Then, Qian et al.’s study introduced a wind-driven hybridized energy harvester (WH-EH) designed to enhance natural disaster monitoring through self-powered wireless sensor networks (WSNs) [98]. This advanced device integrates a TENG and electromagnetic generators (EMGs) with a flexible solar cell, effectively harnessing wind and solar energy (Figure 10b). The WH-EH’s rotator, driven by wind, combines negative polymer triboelectrification materials with eighteen strategically placed magnets and precision-manufactured coils. This design ensures total isolation from harsh environmental conditions, maximizing energy utilization efficiency. The WH-EH is notable for its high-voltage, low-current TENG output, complementing the EMGs’ differing performance to deliver outstanding power across a broad frequency range. Capable of lighting hundreds of LEDs and powering small electronics, its quick capacitor charging ability was demonstrated in experimental tests. The device’s practical applications are vast, including temperature sensors for forest fire detection, vibration sensors for earthquake monitoring, and wireless transceivers for spreading alarm information.

Meanwhile, Dudem et al. have innovatively designed a hybrid energy cell, integrating hierarchical nanoarchitectured/microarchitectured polydimethylsiloxane (HNMA-PDMS) films (Figure 10c) [99]. These films, characterized by their nano/micro dual-scale architectures, namely, nanonipples on inverted micropyramidal arrays, are pivotal in the multifaceted energy-harvesting capabilities of the device. Employing a cost-effective soft imprint lithography process, the HNMA-PDMS films are replicated, combining the functions of a triboelectric layer in TENGs, an antireflection layer in dye-sensitized solar cells (DSSCs), and a self-cleaning surface. The device’s effectiveness is amplified by the large contact area provided by the PDMS film’s hierarchical structures, enhancing the TENG’s performance. Remarkably, the HNMA-PDMS/ITO/PET structure of the device, with its high transmittance, contributes to the transparency of the TENGs. By integrating the HNMA-PDMS/ITO/PET with zinc oxide nanowires into DSSCs, the device becomes a hybrid energy cell capable of converting mechanical, solar, and wind energies into electricity, either simultaneously or independently.

Finally, Wang et al.’s research in urban environmental energy harvesting introduces a versatile and innovative hybridized nanogenerator [100]. This device combines a solar cell (SC) and a TENG to efficiently scavenge solar and wind energies, either individually or simultaneously (Figure 10d). Specifically engineered for urban deployment, these nanogenerators boast a compact footprint of approximately 120 mm × 22 mm, making them ideal for large-scale installation on city building rooftops. The SC component, under optimal conditions, can generate a maximum output power of about 8 mW, while the TENG delivers up to 26 mW. This remarkable efficiency is further enhanced by a transformer used to reduce the TENG’s impedance, thereby achieving effective impedance matching with the SC. This hybrid system outperforms individual SC or TENG units in both output current and charging capabilities, significantly enhancing its utility.

## 5. Conclusions and Perspectives

### 5.1. TENG Energy Collection in Mixed Energy Collection

#### 5.1.1. Increase in Output Power

The quest to increase TENG output power in the future hinges on several promising avenues of research and development. Material innovation will play a crucial role, particularly in exploring new triboelectric materials with enhanced charge affinity and lower wear rates. Nanoengineering techniques can be employed to create surface textures that optimize the contact electrification process, thereby maximizing energy conversion efficiency. For instance, nanostructured polymers or composite materials could significantly increase the effective contact area and surface charge density, leading to higher power outputs.

Another promising direction is the integration of advanced functional materials, such as piezoelectric or ferroelectric layers, within TENG structures. These materials can augment triboelectric charging, leading to a synergistic effect that boosts the overall power generation. Furthermore, the exploration of smart, responsive materials that can adapt to environmental changes—such as temperature, humidity, and pressure—will enable TENGs to operate optimally under varying conditions.

In addition to material innovations, advancements in TENG design and architecture will be pivotal. This includes exploring multilayer TENG configurations and integrating TENGs into various structures and objects, such as clothing, buildings, and vehicles, to harness energy from everyday activities and movements. The development of scalable TENG arrays, capable of covering larger areas, will also be instrumental in enhancing the overall power output.

#### 5.1.2. Increase in Stability

Enhancing the stability of TENGs for future applications necessitates a focus on both material durability and environmental resilience. Research should prioritize the development of materials that can withstand mechanical stress, environmental exposure, and long-term wear and tear without significant degradation in performance. This includes investigating robust yet flexible materials, such as advanced polymers and composites, that can endure repeated compressions, stretches, and abrasions.

A particularly promising area is the incorporation of self-healing materials into TENGs. Such materials can repair themselves after damage, maintaining their triboelectric properties over time. This self-healing capability can be achieved through various mechanisms, including reversible chemical bonds or microcapsule-based systems that release healing agents upon damage.

The stability of TENGs in diverse environmental conditions is another critical aspect. Developing TENGs that are resistant to moisture, temperature fluctuations, and UV exposure is essential for their longevity, especially in outdoor or harsh environments. Protective coatings and encapsulation techniques can play a significant role in shielding TENGs from environmental factors. Additionally, the integration of moisture-absorbing or hydrophobic layers can prevent performance degradation due to humidity.

Finally, advancements in the electrical components of TENG systems, such as more durable electrodes and improved insulation materials, will contribute to the overall stability and longevity of TENGs. Coupled with intelligent monitoring systems that can detect and respond to performance issues, these advancements will ensure that TENGs remain a reliable and stable source of energy for a wide range of applications.

### 5.2. Solar Energy Collection in Mixed Energy Collection

#### 5.2.1. Increase in Output Power of Solar Energy Collection

To significantly increase the output power of solar energy collection in the future, a multifaceted approach focused on material science, technological innovation, and system integration will be essential. The development of next-generation solar cells, such as advanced perovskite solar cells, holds immense promise. These cells not only are more efficient at converting sunlight into electricity but also offer the potential for tandem solar cell structures, where layers of different materials are used to capture a broader spectrum of sunlight. This approach could drastically increase the power conversion efficiency beyond the current limits of traditional silicon-based cells.

Another key area of focus is the utilization of nanotechnology to enhance light absorption and reduce energy losses in solar cells. For instance, the use of quantum dots and plasmonic nanoparticles can increase the absorption of solar radiation and facilitate the efficient use of photons. Additionally, developments in photon conversion technologies can enable solar cells to harness energy from low-energy photons, which are abundant but currently underutilized in solar energy harvesting.

Improving the architectural design of solar panels is also vital. Innovations such as bifacial solar panels, which can capture sunlight from both sides, and solar tracking systems, which adjust the panels’ orientation to follow the sun’s path, can significantly enhance the total energy output. Integrating solar panels into building materials, known as building-integrated photovoltaics (BIPV), can also increase the surface area available for energy collection without requiring additional land use.

#### 5.2.2. Increase in Stability of Solar Energy Collection System

Enhancing the stability of solar energy collection systems is crucial for ensuring long-term, reliable performance. Future advancements in this area will likely involve the development of more durable solar cell materials and protective coatings. For instance, research into more stable perovskite compositions, which are less susceptible to degradation from environmental factors like moisture and heat, will be pivotal. This includes exploring new perovskite formulations with intrinsic stability and developing advanced encapsulation techniques to protect the cells from external elements.

Improving the environmental resilience of solar panels will also involve innovative designs and materials that can withstand extreme weather conditions, such as high winds, hail, or extreme temperatures, without significant degradation in performance. For example, the development of flexible, lightweight, and yet robust solar panel materials can contribute to greater durability, especially in harsh environmental conditions.

In addition, advancements in self-cleaning technologies for solar panels can play a significant role in maintaining efficiency over time. The implementation of hydrophobic coatings or the integration of nanomaterials that repel dust and water can help keep the solar panels clean, ensuring maximum light absorption and reducing the need for maintenance.

Finally, the stability of solar energy systems can be further enhanced by integrating advanced energy storage solutions. Developing more efficient and longer-lasting batteries or exploring new storage technologies like hydrogen fuel cells can provide a stable supply of solar energy, even during periods without sunlight. This integration of efficient energy storage with solar panels will be crucial in transitioning toward a more resilient and sustainable solar energy future.

### 5.3. Future Progress in Mixed Energy Collection

#### 5.3.1. Expanding Energy Collection Channels

Expanding the energy collection channels of TENGs in future mixed energy systems is of great importance for enhancing their versatility and overall efficiency. This expansion primarily focuses on integrating TENG technology with various renewable energy sources, thereby broadening its application spectrum. A key area of development is the amalgamation of TENGs with wind energy harvesting. Innovations in TENG design, such as incorporating flexible, wind-responsive materials, can enable the capture of energy from airflows and breezes at varying speeds, effectively turning TENGs into dual-function devices that harness both mechanical and wind energy.

Another promising direction is the integration of TENGs with bioenergy systems. By utilizing biologically derived mechanical movements, such as those from plants or microbial activity, TENGs can convert these natural kinetic energies into electrical power. This integration not only diversifies the energy sources for TENGs but also contributes to more sustainable bioenergy utilization.

Furthermore, exploring the synergies between TENGs and hydrodynamic sources, like tidal and wave energy, presents substantial opportunities. Adapting TENG materials and structures to function in aquatic environments, where they can harness energy from water movements, can significantly increase the range of energy collection. This approach leverages the inherent mechanical energy present in water currents and waves, seamlessly fitting into the operational mechanism of TENGs.

Thermoelectric energy harvesting also offers a complementary avenue for TENGs. By integrating thermoelectric elements into TENG systems, it is possible to harness temperature gradients, prevalent in many environments, to generate electricity. This combination can be particularly effective in scenarios where thermal and mechanical energies coexist, such as industrial processes or natural geothermal areas.

Last, capitalizing on ambient energy sources like RF energy or electromagnetic fields, typically underutilized, can further expand TENGs’ energy collection channels. Incorporating materials or designs within TENGs that respond to these ambient energies can transform them into more comprehensive energy-harvesting systems.

By diversifying the energy collection channels and integrating TENG technology with various renewable sources, future mixed energy systems can achieve a more robust, efficient, and sustainable energy-harvesting capability, aligning with the evolving demands of global energy needs.

#### 5.3.2. Stability Between Different Energy-Harvesting Modules

Improving the stability between different energy-harvesting modules in future mixed energy collection systems will require sophisticated control and management strategies. The development of intelligent energy management systems, utilizing advanced algorithms and machine learning, will be key in optimizing the interaction and energy flow between different modules. These systems can dynamically adjust energy collection and distribution based on real-time data, environmental conditions, and energy demand, ensuring maximum efficiency and stability.

In terms of hardware, the focus will be on developing modular designs that allow for seamless integration and interoperability of different energy-harvesting technologies. This includes standardizing interfaces and electrical components to facilitate easy coupling of solar, wind, TENG, and other energy modules. Research into hybrid energy converters capable of processing energy from multiple sources simultaneously will also contribute to system stability.

Furthermore, advancements in energy storage technologies will play a crucial role in stabilizing mixed energy systems. Integrating next-generation batteries, supercapacitors, and even emerging technologies like solid-state batteries or gravity-based storage systems can provide buffer capacity to manage fluctuations in energy supply from different sources. This storage capability ensures a continuous and stable energy supply, even when individual energy-harvesting modules experience variability in their output.

Finally, enhancing the stability of mixed energy systems will also involve improving the durability and resilience of individual modules. Research into materials and designs that can withstand environmental stressors, such as temperature fluctuations, moisture, and mechanical wear, will ensure long-term stability and reduce maintenance needs. By addressing these technical and systemic challenges, future mixed energy systems can achieve a harmonious and stable operation, unlocking their full potential in providing sustainable and reliable energy solutions.

As a conclusion, the introduction of functional materials such as perovskite can further increase the energy-harvesting efficiency of TENGs and broaden the application fields, and integrating the TENG with other types of energy harvesters such as solar energy systems is a highly attractive pathway to conquer challenges such as environmental impacts, weather constraints, instability, low utilization, etc. However, additional complexities have also been introduced at the same time, such as system integration, optimization, and energy management. And the efficiency of the hybrid system depends on the effective connection of solar and mechanical energy collection mechanisms, which is a technically demanding task. There is still a long way to go in scientific research.

## Figures and Tables

**Table 1:** The performance of perovskite-based TENGs.

Materials	Voltage	Current Density	Power Density	Ref.
CsPbBr_3_/nanoplatinum	273 V	30.3 mA cm^−2^	1295.5 mW m^−2^	[66]
Co(OH)(CO_3_)_0.5_/Pt/CsPbIBr_2_	243 V	3.1 μA cm^−2^	/	[67]
CsPbCl_3_	258 V	/	3.06 W m^−2^	[68]
CsPbBr_2.6_I_0.4_	192 V	167 μA cm^−2^	1.2 W m^−2^	[69]
CsPbBr_3_	240 V	4.13 μA cm^−2^	3.31 W m^−2^	[70]
AgNWs/(Bi_0.5_Na_0.5_)TiO_3_-BaTiO_3_ (Mn-BNT-BT)	2172 V	/	47.3 W m^−2^	[71]
Organic–inorganic hybrid perovskite materials (PTFE/PA-MAI/PbI_2_ ratio of 2)	979 V	/	24 W m^−2^	[74]
n-type Cs_0.175_FA_0.750_MA_0.075_Pb(I_0.880_Br_0.120_)_3_ (CsFAMA) and p-type PEDOT	0.8 V	11 μA cm^−2^	/	[75]

**Table 2:** Perovskite-based hybrid energy collection system.

Structure A	Structure B	Achivements/Applications	Ref.
TENG-powered OLED	Solar cell-powered PPD	Substantial data transmission	[84]
Electromagnetic-TENG units	A flexible solar cell	Wireless transmission over a distance of about 100 cm	[85]
PA-TENG	/	1.17 W m^−2^ power output. Maintain higher transparency and power output during both rainy and sunny days	[86]
Ionogel-based TENG	/	Energy harvesting from water droplets	[87]
CsPbBr_3_ quantum dots/PDMS film-TENG	Si solar cell	Harvesting both solar and rain energy, the PCE increases from 18.47% to 20.16%	[89]
Droplet TENG	Photovoltaic cell (PVC)	Harvesting energy from both light and liquid droplets. Achieving a maximum peak power density of 3.71 mW m^−2^ and an output energy density of 15.36 mJ m^−2^.	[91]
Spherical TENGs	A solar cell	Harvesting energy from wind, rain, and sunlight. Achieving an average power output of 5.63 mW	[95]
